# Altered Functions and Interactions of Glaucoma-Associated Mutants of Optineurin

**DOI:** 10.3389/fimmu.2018.01287

**Published:** 2018-06-06

**Authors:** Ghanshyam Swarup, Zuberwasim Sayyad

**Affiliations:** CSIR-Centre for Cellular and Molecular Biology, Hyderabad, India

**Keywords:** optineurin, glaucoma, autophagy, NF-κB, TBK1, vesicle trafficking, CYLD, Rab8

## Abstract

Optineurin (OPTN) is an adaptor protein that is involved in mediating a variety of cellular processes such as signaling, vesicle trafficking, and autophagy. Certain mutations in OPTN (gene *OPTN*) are associated with primary open angle glaucoma, a leading cause of irreversible blindness, and amyotrophic lateral sclerosis, a fatal motor neuron disease. Glaucoma-associated mutations of OPTN are mostly missense mutations. OPTN mediates its functions by interacting with various proteins and altered interactions of OPTN mutants with various proteins primarily contribute to functional defects. It interacts with Rab8, myosin VI, Huntigtin, TBC1D17, and transferrin receptor to mediate various membrane vesicle trafficking pathways. It is an autophagy receptor that mediates cargo-selective as well as non-selective autophagy. Glaucoma-associated mutants of OPTN, E50K, and M98K, cause defective vesicle trafficking, autophagy, and signaling that contribute to death of retinal ganglion cells (RGCs). Transgenic mice expressing E50K-OPTN show loss of RGCs and persistent reactive gliosis. TBK1 protein kinase, which mediates E50K-OPTN and M98K-OPTN induced cell death, is emerging as a potential drug target. Autoimmunity has been implicated in glaucoma but involvement of OPTN or its mutants in autoimmnity has not been explored. In this review, we highlight the main functions of OPTN and how glaucoma-associated mutants alter these functions. We also discuss some of the controversies, such as the role of OPTN in signaling to transcription factor NF-κB, interferon signaling, and use of RGC-5 cell line as a cell culture model.

## Introduction

**Figure 1:**
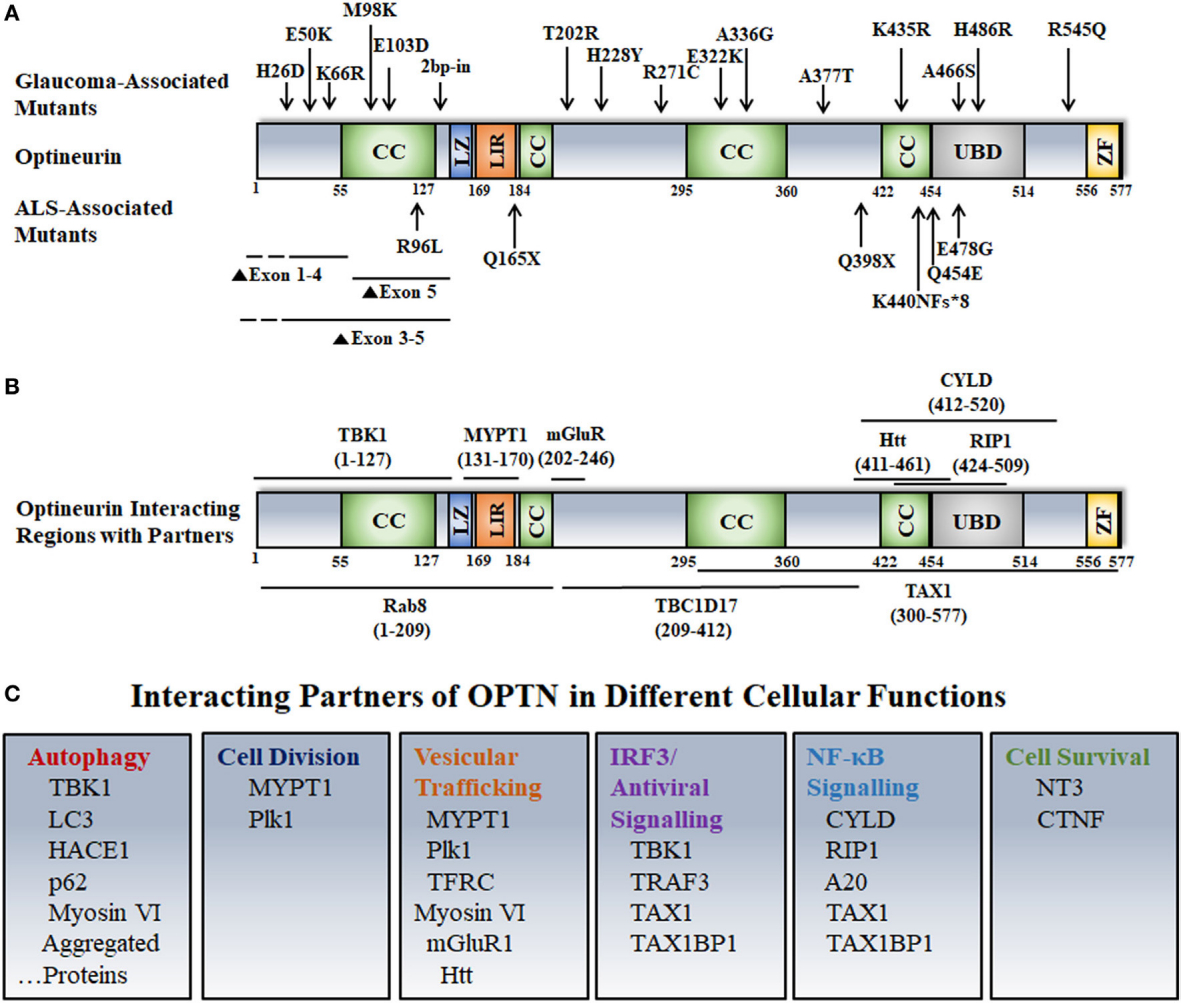
Disease-associated mutations, interactions, and cellular functions of optineurin. **(A)** Various domains and disease-associated mutants of OPTN are shown. Abbreviations: CC, coiled coil; LZ, leucine zipper; LIR, LC3 interacting region; UBD, ubiquitin-binding domain; ZF, zinc finger. **(B)** OPTN-interacting proteins and their binding sites on OPTN are shown. **(C)** Interacting partners of OPTN involved in various cellular functions are depicted.

Optineurin (OPTN) is a multifunctional protein, which mediates various signaling and vesicle trafficking pathways, and autophagy. It is expressed in several types of cells and tissues in mice and humans such as retina, brain, liver, heart, etc. (1–5). Expression of OPTN is induced by cytokines such as interferons and tumor necrosis factor α (TNFα) (6). Human OPTN is a 577 amino-acid protein with several coiled coil domains, an ubiquitin-binding domain (UBD), a zinc finger, and an LC3-interaction region (LIR) (Figure 1). Certain mutations in OPTN are associated with glaucoma, an eye disease that causes progressive irreversible blindness (7). Genetic as well as environmental factors contribute to onset and progression of glaucoma (8–10). Increased intra-ocular pressure (IOP), family history, and old age are some of the risk factors for primary open angle glaucoma (POAG), the most prevalent form of glaucoma in adults (11). About two-third of POAG is associated with increased IOP, which generally occurs due to obstruction of outflow of aqueous humor in trabecular meshwork. About one-third of POAG patients have IOP in the normal range [normal tension glaucoma (NTG)], but vision loss occurs due to degeneration of retinal ganglion cells (RGCs) (12). In glaucoma, impaired vision occurs due to degeneration of RGCs and their exons in the optic nerve head. The function of RGCs is to receive the signal from photoreceptor cells *via* the bipolar and amacrine cells, and to transmit this visual signal, in the form of action potential, to the brain *via* optic nerve (13). RGCs have different sizes and connections, but they all have a long axon, and these axons form the optic nerve (14).

## Mutations in OPTN Cause Glaucoma and Amyotrophic Lateral Sclerosis (ALS)

In a study of families affected with NTG, Rezaie et al. in 2002 found that mutations in OPTN are associated with this disease in 16.7% of the families (1). Later on, certain mutations in OPTN were found to be associated with ALS, a fatal motor neuron disease (15). Glaucoma-associated mutations of OPTN are mostly missense mutations, whereas ALS-associated mutations include deletions, missense, and nonsense mutations. In general, glaucoma-associated mutations are not associated with ALS with one exception, a two-base pair insertion in exon 6, which is very rare. OPTN was the first gene wherein mutations were found to be associated with NTG (1). Amplification of *TBK1* gene is associated with NTG although no mutations have been reported so far (12, 16). Several missense mutations of OPTN have been reported that are associated with glaucoma, such as E50K, H26D, H486R, E322K, etc. (7). In a large family, the E50K mutation segregates with the disease in individuals over 30 years of age, suggesting, therefore, that this mutation causes glaucoma (1). Such evidence is not available with other mutations of OPTN. In sporadic cases of NTG, OPTN mutations account for about 1% or less of the cases. M98K polymorphism was initially found to be associated with NTG (1). However, subsequent studies have revealed that M98K polymorphism is associated with glaucoma in Asian populations but not in Caucasian populations (17–26).

## Cellular Functions of OPTN

### Vesicle Trafficking and Maintenance of the Golgi Architecture

Optineurin is localized predominantly in the cytoplasm, but it is also seen in the Golgi complex, various membrane vesicles, and autophagosomes (1, 27–30). Upon treatment of cells with high level of H_2_O_2_, it can move into the nucleus (5). However, the function of OPTN in the nucleus is not known. Knockdown of OPTN results in breakdown of the Golgi structure, suggesting its role in stabilization of the architecture of the Golgi (31–33). OPTN interacts with several proteins involved in vesicle trafficking such as Rab8, Huntingtin, myosin VI, TBC1D17, transferrin receptor (TFRC), etc. (7, 30, 34–36). OPTN provides a link between Rab8 GTPase and the actin-based molecular motor, myosin VI. It also links myosin VI with the Golgi complex (34). OPTN plays a role in exocytosis and also in Rab8-mediated polarized membrane vesicle transport in epithelial cells (31).

Optineurin interacts with the activated GTP-bound form of Rab8 and, therefore, it is considered as an effecter of Rab8 that mediates some of the functions of Rab8 (36). The interaction of OPTN with the inactive GDP-bound form of Rab8 is very weak (36, 37). Rab8 is involved in several vesicle trafficking functions including endocytic trafficking and recycling of TFRC, a protein involved in iron uptake through receptor-mediated endocytosis (38). Iron binds with transferrin, and then transferrin with bound iron interacts with the TFRC on the outer surface of the cell. Transferrin–TFRC complex is endocytosed primarily through clathrin-dependent endocytosis and reaches early endosomes where iron is released in the endosome due to acidic environment (39). The iron is then transported out of endosome into the cytoplasm through iron transporters and transferrin–TFRC complex is recycled back to the plasma membrane either directly from early endosome or through the recycling endosome (38). Knockdown of OPTN as well as Rab8 leads to slower trafficking of TFRC-positive endosomes to recycling endosomes. In addition, Rab8 as well as OPTN are involved in recycling of TFRC-positive vesicles to the plasma membrane (35, 37). Activated Rab8 forms tubules emanating from endocytic recycling compartment, and these tubules facilitate movement of TFRC-positive vesicles to the plasma membrane (35, 37). OPTN is not only an effector of Rab8, it is also a negative regulator of Rab8 activity (37). TBC1D17, a Rab GTPase-activating protein (GAP) was identified as OPTN interacting protein by yeast two-hybrid screen (40). Central region of OPTN interacts with TBC1D17, whereas N-terminal region interacts with Rab8. TBC1D17 does not interact with Rab8 directly but requires OPTN for this interaction and also for inactivation of Rab8 (37). After binding with activated Rab8, OPTN recruits TBC1D17, which inactivates Rab8 resulting in inhibition of TFRC recycling. Thus, OPTN acts as an effector of Rab8 and also as an adaptor protein to bring together Rab8 and its GAP. This model of Rab8 regulation by an effecter protein easily explains transient nature of Rab activation (37).

### Autophagic Functions of OPTN

Autophagy and ubiquitin proteasome system are the two major protein degradation pathways in the cell. Macro-autophagy (hereafter referred to as autophagy) is a quality control mechanism used by the cell to remove damaged and aggregated proteins, damaged organelles, invading bacteria, etc., through degradation in lysosomes (41). Autophagy occurs at low level under basal condition to maintain homeostasis, but it can be induced by stress such as nutrient starvation or accumulation of cargo to be degraded. Upon induction of autophagy, a specialized double-membrane structure known as isolation membrane or phagophore is formed that matures into autophagosome, which recruits the cargo to be degraded. Autophagosome then fuses with lysosome to form autolysosome where degradation of cargo takes place. Autophagosomal protein LC3 serves as a useful marker for autophagosome and the level of its lipidated form, LC3-II is often used as a measure of autophagy (42). LC3-II serves crucial functions during autophagy, such as expansion and closure of phagophore to form autophagosome, and recruitment of cargo. Recruitment of cargo is mediated by a group of proteins known as autophagy receptors that link LC3-II with the ubiquitinated cargo. Ubiquination of cargo very often serves as a signal for identification and recruitment by autophagy receptors to the autophagosome (43). OPTN was identified as an autophagy receptor, which can directly interact with LC3 through LIR and also with ubiquitinated cargo through its UBD located in the C-terminal region (29). Phosphorylation of OPTN at S177 in the LIR increases its binding to LC3 and promotes autophagy-mediated clearance of cytosolic bacteria and mutant protein aggregates. UBD of OPTN recognizes LUBAC-synthesized linear ubiquitin chains on Samonella, which facilitates their clearance by autophagy (44).

Optineurin, along with other autophagy receptors, is involved in movement of autophagosomes for fusion with lysosomes. This movement of autophagosomes, like movement of membrane vesicles, is mediated by a molecular motor protein, myosin VI that moves on actin cytoskeletal tracks. Myosin VI directly interacts with OPTN and this interaction is involved in movement and fusion of autophagosome with lysosome (45).

Recently, another function of OPTN was discovered where it is involved in the maturation of phagophore to form autophagosome. Optn^−/−^ mouse embryonic fibroblasts (MEFs), generated by homologous recombination, show decreased level of LC3-II and lower number of autophagosomes and autolysosomes (46). However, the number of phagophores is not reduced but increased in Optn^−/−^ MEFs, suggesting thereby that Optn is required for efficient maturation of phagophore into autophagosome, and also for LC-II formation. Conjugation of LC-I with the lipid phosphatidylethanolamine to form LC3-II occurs by a ubiquitination-like conjugation reaction, which is mediated by an E3-ligase like enzyme, the Atg12-5-16L1 complex. Optn interacts with Atg5 and facilitates recruitment of the Atg12-5-16L1 complex to the phagophore to promote maturation of phagophore into autophagosome. A phosphodefective mutant of Optn, S177A, unlike normal OPTN, does not promote autophagosome formation, suggesting that phosphorylation of OPTN at S177 is involved in autophagosome formation (46). Optn is also involved in autophagosome formation in retinal cells (47–50). OPTN interacts with Rab1a, and it has been suggested that this interaction is required for autophagosome formation in Neuro2a cells (51). However, direct evidence for this conclusion is lacking and the mechanism by which Rab1a–Optn interaction promotes autophagosome formation has not been investigated.

Optineurin and NDP52 are the autophagy receptors that are involved in clearance of damaged mitochondria by autophagy (mitophagy) (52). Damaged mitochondria get ubiquitinated that recruit autophagy receptors including OPTN, which mediate delivery to autophagosomes (53–55). All the studies on involvement of OPTN in mitophagy have been carried out using chemicals to damage mitochondria. The role of OPTN in basal mitophagy or physiologically relevant mitophagy, is yet to be established using animal models.

### OPTN as a Negative Regulator of NF-κB Signaling: Fact or Artifact?

NF-κB is an inducible transcription factor that is kept in the inactive state by IκB inhibitory proteins by sequestering in the cytoplasm. Upon induction by inducers, such as cytokines, it moves into the nucleus where it activates transcription of genes involved in cell survival and cell division, inflammation, immune response, etc. (56). Deregulation of NF-κB is involved in several disorders including neurodegeneration and glaucoma (57). OPTN is a negative regulator of basal as well as TNFα-induced NF-κB activity (58, 59). It was proposed that OPTN competes with NEMO for binding to polyubiquitinated RIP leading to inhibition of IKK and NF-κB activation (59). In this process, UBD of OPTN binds with Lys63-linked polyubiquitin chains on RIP. However, this mechanism does not explain regulation of basal NF-κB activity by OPTN (59). Subsequent work has shown that OPTN interacts with the deubiquitinase CYLD, a negative regulator of NF-κB that deubiquitinates RIP (40). OPTN acts as an adapter protein to bring together an enzyme (CYLD) and its substrate RIP to regulate NF-κB activity (60). Knockdown of OPTN in unstimulated cells leads to accumulation of ubiquitinated RIP that leads to increased basal NF-κB activity. OPTN is required for CYLD-dependent deubiquitination of RIP and also for CYLD-dependent inhibition of TNFα-induced by NF-κB activity (60). This mechanism of by NF-κB regulation by OPTN is supported by the findings, which show that a glaucoma-associated mutant of OPTN, H486R, is defective in interaction with CYLD and is unable to inhibit TNFα-induced NF-κB activation (60). Interestingly, OPTN gene expression is regulated by NF-κB, which binds to a site in the OPTN promoter (58). Thus, OPTN, a negative regulator of NF-κB is induced by NF-κB, making a feedback loop.

Interaction of OPTN with CYLD is also involved in regulating NF-κB activation mediated by toll-like receptor signaling induced by lipopolysaccharide (LPS), and interleukin-1 receptor (IL1R) signaling (61). OPTN interacts with IRAK1 (IL1R-associated kinase-1) that mediates NF-κB activation in response to IL1R activation. OPTN negatively regulates IRAK-1 mediated NF-κB activation possibly by facilitating CYLD-dependent deubiquitination of TRAF6. Unlike wild type OPTN, the H486R mutant is unable to inhibit LPS- or Il1β- or IRAK1 overexpression-induced NF-κB activation (61). During osteoclast differentiation, OPTN negatively regulates RANKL-induced NF-κB activation and osteoclast differentiation. Interaction of OPTN with CYLD is proposed to be involved in this regulation (62).

It has been suggested that TNFα-induced NF-κB regulation by OPTN is an artifact of *in vitro* experiments, because this was not seen in bone marrow-derived macrophages (BMDM) obtained from Optn mutant mice (63, 64). NF-κB activation is controlled by several mechanisms and these mechanisms may be altered in mutant mice, which may explain lack of TNFα-induced NF-κB activation in BMDM. Another possibility is that in cells lacking normal Optn, some other protein may act as an adaptor to facilitate CYLD-dependent deubiquitination of RIP. It is also possible that Optn has cell type specific functions. It seems unlikely that all the siRNAs or shRNAs (but not the control siRNas/shRNAs) used in different cells to knockdown OPTN produce artifact to give increase in NF-κB activity (58–60, 65). OPTN knockout Hela cells generated using CRISPR/Cas9 technique also showed enhanced TNF-α mediated NF-κB activity (66). Interestingly, bone marrow cells derived from D474N-OPTN mutant mice show increased RANKL-induced NF-κB activity during osteoclast differentiation (62).

### Interferon Signaling and OPTN

Type I interferons are produced in response to viral and bacterial infections as a defense mechanism for eliminating these pathogens (67). Activation of IRF3 by viral or bacterial components is an important step in the induction of IFN β gene expression. TBK1 is one of the kinases that phosphorylates and activates IRF3. Mankouri et al. reported that OPTN is a negative regulator of IFN β expression induced by Sendai virus and dsRNA in HEK cells (68). OPTN recruits CYLD to TBK1, which results in reduced TBK1 activation during antiviral signaling in HeLa cells (69). In contrast to these reports, some other studies have shown that OPTN is a positive regulator of TBK1 activity, IRF3 phosphorylation, and IFN β gene expression in mouse macrophages in response to LPS and poly (I:C) (63, 64, 70). Although the reasons for these discrepancies are not known, it is likely that cell type differences may explain these observations. It has also been suggested that human and mouse OPTN may differ in their interaction with other proteins such as CYLD, which may explain these discrepancies (69, 71).

### Cytoprotective and Other Functions of OPTN

A cytoprotective function of OPTN was proposed to explain pathogenesis of glaucoma, which is impaired by mutations (1). OPTN is required for secretion of neurotrophins, which help in cell survival (72). Role of negative regulation of NF-κB by OPTN in neuronal cell survival has been proposed (65). It is likely that several functions of OPTN contribute to cell survival including autophagy, vesicle trafficking, signaling, etc. Overexpression of OPTN in primary RGCs *in vitro* results in increased mitochondrial biogenesis as seen by increased mitochondrial fission and increased mitochondrial volume density (73). However, the role of endogenous OPTN in mitochondrial biogenesis is yet to be established. A role for OPTN in mitosis has also been proposed (74). However, OPTN knockout mice are born normal.

## Model Systems to Study Glaucoma Pathogenesis

### Animal Models

Some animal models such as DBA/2J mice have been developed to study pathogenesis of high pressure glaucoma (75). However, to study pathogenesis of NTG, these models are not appropriate (75). Glutamate transporter knockout mice have been developed as NTG models, which show loss of RGCs as well photoreceptor cells (76). Transgenic mice expressing E50K-OPTN have been developed, which show glaucoma phenotype such as loss of RGCs, thinning of various cell layers of retina and gliosis (7, 77–79). However, these mice do not show increase in IOP suggesting that these are useful as NTG model. Recently, a knock-in mouse model expressing E50K mutation in Optn has been generated using CRISPER/Cas9 genome editing (7). These mice, unlike E50K transgenic mice, do not show thinning of retina but in homozygous condition these mice show reduction in RGC fiber layer around optic nerve head and glaucomatous cupping.

### Cell Culture Models and the Controversy About the Nature of RGC-5 Cell Line

**Figure 2:**
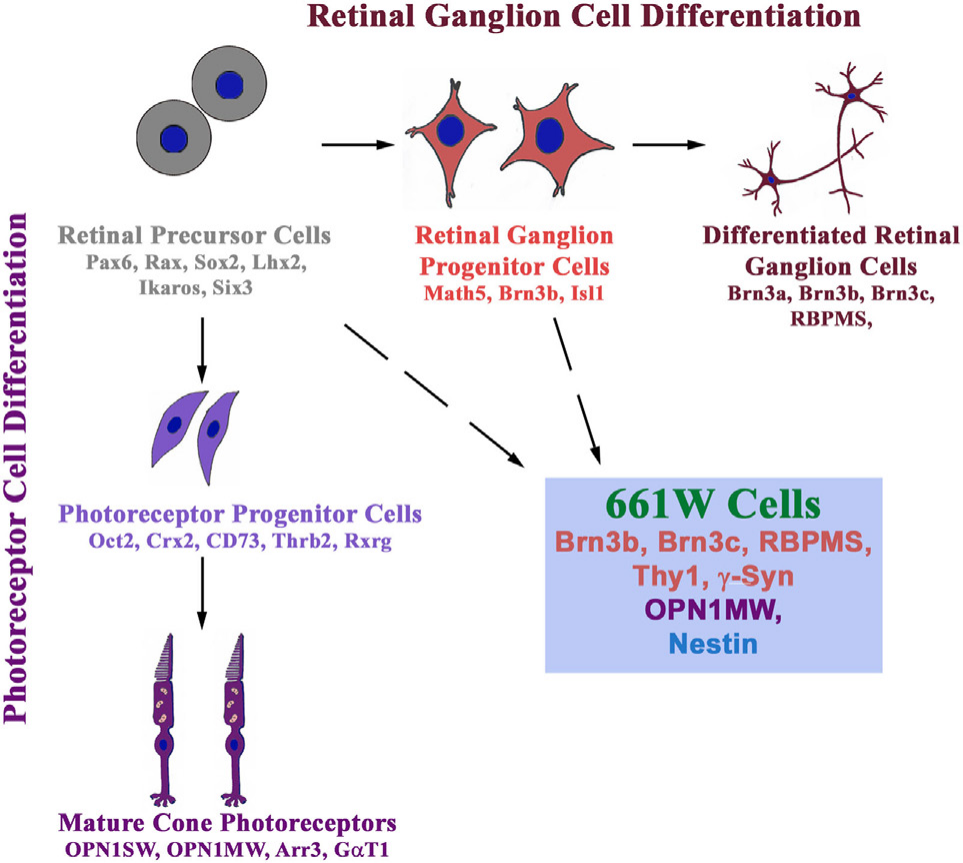
661W is a retinal ganglion precursor-like cell line that shows features of both retinal ganglion and cone photoreceptor cells. These cells are probably derived from either retinal precursor cells (which give rise to various types of cells in the retina) or retinal ganglion progenitor cells, which differentiate into retinal ganglion cells.

Cell culture models are useful to study the molecular mechanisms and functional defects caused by glaucoma-associated mutants. These are also needed for initial screening for cytoprotective compounds, which can be developed as potential drugs for treatment of glaucoma. RGC-5, a retinal ganglion cell line, thought to be of rat origin, was described in 2001, which was shown to express several molecular markers of RGCs such as Thy1, Brn3C, etc. (80). The RGC-5 cell line was later found to be of mouse origin and was probably the same as 661W cell line, described as a cone photoreceptor cell line that was derived from a mouse retinal tumor induced by expression of SV40 T antigen (81, 82). Expression of some markers of RGCs like Thy1, Brn3 family proteins, neuroflaments was not seen in RGC-5 cells by some groups, although some neuronal markers such as β III tubulin, MAP2, MAP1b, etc., were seen (83, 84). On the basis of these and a few other observations, it was suggested that RGC-5 is not an RGC line. In these studies, Brn3 family of transcription regulatory proteins (Brn3a, Brn3b, Brn3c), which are involved in differentiation of RGCs, was not analyzed properly and depended on a single antibody to detect three different gene products by immunostaining of cells. The specificity of this commercial Brn3 antibody was not tested by immunoblotting (84). Interestingly, 661W as well as RGC-5 cells can be differentiated into cells with prominent neuronal morphology (85). Recently, both 661W and RGC-5 cell lines have been re-characterized (86). Both these cell lines express several molecular markers of RGCs such as Rbpms, Thy1, Brn3b, Brn3c, γ-Synuclein, β III tubulin, and NeuN. In addition, these cell lines express nestin, a neuronal precursor marker. But these cells also express a cone specific marker, Opn1mw at low level. Treatment of 661W as well as RGC-5 cells with staurosporine leads to differentiation into RGC-like cells, which was accompanied by increase in the level of several RGC marker proteins (86). These observations suggest that 661W cells (as well as RGC-5 cells) are RGC precursor-like cells, which have features of both retinal ganglion and cone photoreceptor cells (Figure 2).

Interestingly, 661W cells show induction of cell death by two glaucoma-associated mutants of OPTN but not by wild-type OPTN or by an ALS-associated mutant E478G (86). In contrast, expression of E478G-OPTN, but not E50K or M98K, induces cell death by apoptosis in NSC34 cells, a motor neuron cell line used as a cell culture model for ALS (86). This RGC-like property of selective induction of cell death by glaucoma-associated mutants of OPTN provides further support to the suggestion that the 661W cells are RGC precursor-like cells. Therefore, these cells can be used for exploring the molecular mechanisms of cell death and other functional defects caused by glaucoma-associated mutants of OPTN. In addition, these cells can also be used for screening or testing of compounds to prevent cell death as seen by the effect of various chemicals that inhibit E50K-OPTN or M98K-OPTN-induced cell death.

Since RGC-5 cells, like 661W cells, express several markers of RGCs, these cells can also be used for exploring the molecular mechanisms of cell death induction and cytoprotection studies. However, we recommend the use of 661W cells because of previous controversy with RGC-5 cells. We also suggest that every user should test several markers in these cell lines because cell lines can change properties over a period of time. Some of the cytoprotection studies carried out previously with 661W cells as photoreceptor cells need to be re-interpreted because these may have relevance to RGCs.

Induced pluripotent stem cells (iPSCs) derived from cells taken from a glaucoma patient carrying E50K-OPTN and a normal control, have been prepared. These cells can be differentiated into RGC-like cells by using available differentiation protocols (78). These cells are likely to be useful to study functional defects caused by mutation and to understand molecular mechanisms of pathogenesis of glaucoma.

## Altered Interactions and Function of OPTN Mutants

### E50K-OPTN Shows Altered Interactions With Cellular Proteins

E50K mutation affects interaction of OPTN with several proteins such as TBK1, Rab8, TBC1D17, and TFRC (7, 30, 40, 64, 87–89). These altered interactions are likely to affect the function of interacting proteins, which possibly contributes to RGC death and glaucoma pathogenesis. This mutation also alters the oligomeric state of OPTN, which would be expected to alter its function in the cell (90, 91).

### E50K-OPTN Induces RGC Death and Glaucoma Pathogenesis

The effect of E50K-OPTN on cell survival and other functions has been studied using cell culture as well as transgenic mouse model (77–79, 86, 92). Studies using various cell lines showed that E50K mutant induces apoptosis-like cell death in retinal cells (RGC-5 and 661W) but not in some other cell lines tested, including two neuronal cell lines, IMR-32, and NSC34 (86, 92). These observations suggest that E50K mutant causes glaucoma possibly by directly inducing the death of RGCs. Transgenic mice expressing E50K-OPTN show loss of RGCs and thinning of most of the cell layers of retina. E50K transgenic mice also show reactive gliosis (78).

### E50K-OPTN Impairs Rab8-Mediated Vesicle Trafficking

The E50K mutant forms vesicle-like structures in various cells, which are much larger in size than those formed by WT OPTN. Formation of large vesicles indicates a block in vesicle trafficking (27). The E50K-OPTN vesicles are mostly positive for TFRC, which is often used as a maker for endosomes. Detailed analysis has revealed that E50K mutant impairs endocytic trafficking and recycling of TFRC, causing its accumulation in endocytic vesicles, which in turn results in depletion of TFRC on the cell membrane that leads to reduced transferrin uptake (27, 28). Molecular mechanism of this defective TFRC recycling caused by E50K has been investigated. Rab8 GTPase and its effecter OPTN regulate endocytic trafficking and recycling of TFRC (35, 37). Upon binding with activated Rab8, OPTN recruits the GAP protein TBC1D17 to inactivate Rab8 (37). The E50K mutant recruits TBC1D17 more efficiently, causing enhanced inactivation of Rab8 that results in impaired Rab8-mediated TFRC recycling. This defective TFRC recycling can be reversed by knockdown of TBC1D17 or by using a catalytic mutant of this GAP protein (37). The E50K-induced death of retinal cells can be partly reversed by knockdown of TBC1D17 and also by co-expression of TFRC. These observations suggest that TBC1D17 mediated impairment of TFRC recycling contributes to E50K-OPTN-induced apoptosis of retinal cells (47).

The E50K mutant has lost the ability to interact with activated Rab8. This was seen in yeast two-hybrid assay, which measures direct interaction and also by a fluorescence based assay in mammalian cells (37, 77). However, the E50K mutant shows more complex formation with Rab8 and TFRC in immunoprecipitation assays (37). Thus, it appears that in a multimolecular complex containing Rab8, OPTN, TFRC, and TBC1D17 the direct interaction between Rab8 and E50K is impaired but indirect interaction is increased, which possibly results in functional positioning of the molecules in such a way that results in enhanced inactivation of Rab8 by TBC1D17 (37).

### E50K-OPTN Impairs Autophagy

Expression of E50K-OPTN increases the level of LC3-II in RGC-5 cells and also in the retina of rats expressing E50K-OPTN (47, 50, 93). Transgenic mouse models expressing E50K-OPTN also showed increased level of LC3-II in the retina (73). Increased level of LC3-II could be either due to an increase in autophagy or due to a block in autophagy (42, 94). Using a fluorescence reporter, mCherry-GFP-LC3b to measure autophagy flux, it was found that expression of E50K in RGC-5 cells results in lower number of autophagosomes as well autolysosomes during starvation-induced autophagy (47). If there is a block in autophagy upon expression of E50K-OPTN, then an inducer of autophagy would be expected to rescue E50K-OPTN induced death of retinal cells. Treatment with an autophagy inducer rapamycin reduced apoptosis induced by expression of E50K-OPTN in RGC-5 and 661W cells and also in RGC layers of rat eyes expressing E50K-OPTN (47, 93). This suggests that E50K-OPTN induced increase in LC3-II level in transgenic mice is likely to be due to a block in autophagy that occurs after the formation of LC3-II and autophagosomes.

E50K-induced block in autophagy was mediated partly by the GAP protein, TBC1D17, as shown by the effect of knockdown of TBC1D17 on autophagy. E50K-induced death of retinal cells was partly inhibited by knockdown of TBC1D17 or by expressing its catalytic mutant. TBC1D17 inhibits autophagy through its catalytic activity although the target Rab involved in autophagy is yet to be identified (47).

### Other Alterations Induced by E50K-OPTN

**Figure 3:**
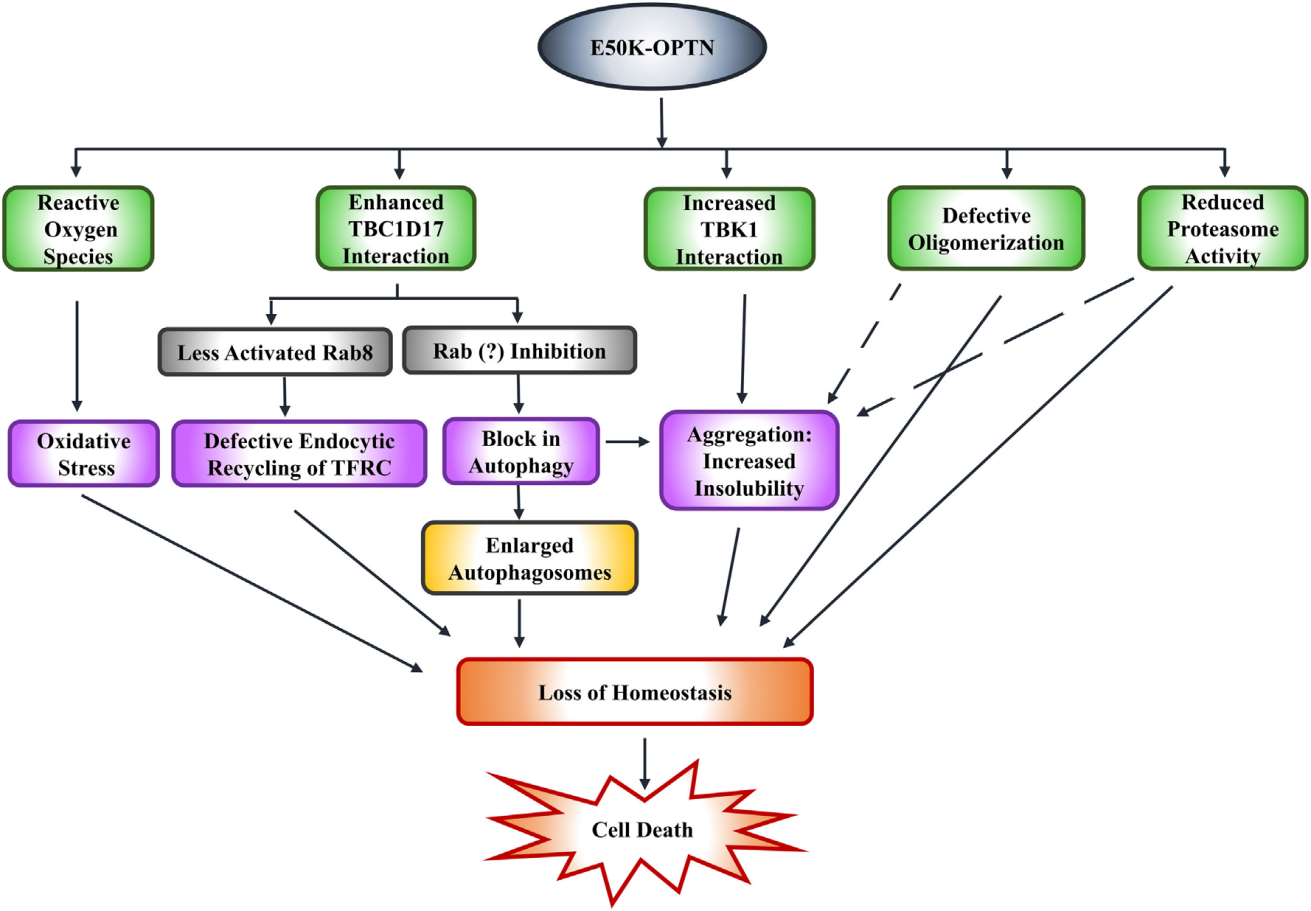
E50K-optineurin induces death of retinal cells by various mechanisms that may contribute to glaucoma pathogenesis.

Expression of E50K-OPTN in HEK cells results in formation of insoluble E50K aggregates. Formation of these aggregates and reduced level of E50K-OPTN in soluble fraction is also seen in iPSC-derived neural cells from an NTG patient. Formation of these E50K aggregates can be prevented by high level of TBK1 inhibitor BX-795 (78). Whether this formation of aggregates by E50K-OPTN is pathogenic or a cytoprotective mechanism used by the cell to reduce toxicity, is not clear. Expression of E50K in retinal cells was shown to increase ROS that mediate cell death. This cell death can be prevented by antioxidants in 661W as well as RGC-5 cells (86, 92). Transgenic mice expressing E50K also show increased ROS levels possibly due to alterations in the levels of certain mitochondrial proteins. It was suggested that E50K-OPTN causes mitochondrial degradation and mitophagy (73). Thus, it appears that E50K mutation alters several functions of OPTN, which contribute to cell death associated with glaucoma pathogenesis (Figure 3).

Transgenic mice expressing E50K-OPTN show differential expression of long non-coding RNAs and micro RNAs, but significance of these changes for RGC death and glaucoma pathogenesis is yet to be explored (95, 96). Expression E50K-OPTN in RGC-5 cells results in reduced expression of miR-9, but its role in E50K-induced cell death has not been explored (97).

### M98K-OPTN Induces Autophagy-Dependent Retinal Cell Death

In the first study of families affected with NTG where OPTN mutations were observed, it was reported that M98K polymorphism was associated with glaucoma (1). Later on, several groups have investigated the association of this polymorphism with glaucoma, and it appears that M98K polymorphism is associated with glaucoma in Asian populations but not in Caucasian populations (17–26). This indicates that M98K polymorphism cooperates with some other genetic alteration to induce glaucoma. Functional alterations in OPTN caused by this polymorphism have been investigated by expressing it in various cell lines. Expression of M98K-OPTN induces significantly more cell death than wild type OPTN in RGC-5 as well as 661W cells but not in several other cell lines, including two neuronal cell lines IMR32 and NSC34 (48, 86). Surprisingly, the apoptotic cell death induced by M98K and E50K occurs by different mechanisms. While E50K-induced cell death is inhibited by antioxidants and antiapoptotic protein BCl2, the M98K- induced cell death is not inhibited by these agents (48, 92). M98K-induced cell death in RGC-5 as well as 661W cells is inhibited by knockdown of Atg5, a protein required for autophagy, and also by chloroquine, an inhibitor of autophagy (48, 86). Expression of M98K in RGC-5 cells results in increased number of autophagosomes as well as autolysosomes, showing enhancement of autophagy. M98K-induced autophagy leads to degradation of TFRC, which results in death of RGC-5 cells. M98K-induced cell death is prevented when TFRC function is restored either by blocking its degradation by chemical inhibitors of autophagy or by co-expression of TFRC by transfection. Supplementation with iron in the growth medium also partly protects from M98K-induced cell death. M98K-OPTN engages Rab12 GTPase to induce autophagy-dependent degradation of TFRC and cell death (48). Thus, it appears that TFRC function of iron homeostasis is critical for survival of RGCs. However, we cannot rule out the possibility that TFRC performs some other function that is also essential for cell survival.

**Figure 4:**
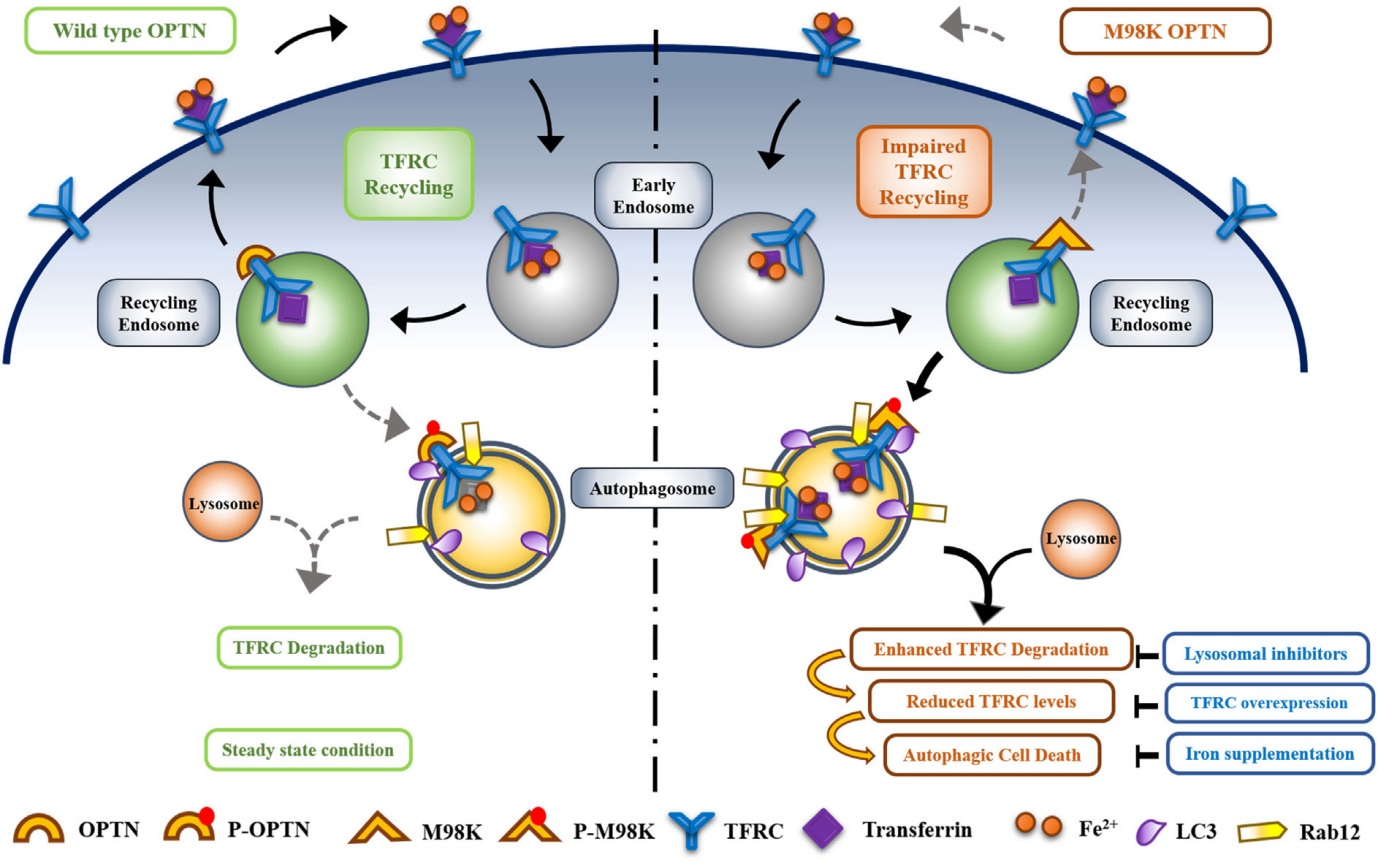
Optineurin (OPTN) acts as a molecular switch that maintains a balance between transferrin receptor (TFRC) recycling and autophagy in retinal cells. M98K mutation in OPTN alters this balance leading to enhanced autophagy that in turn results in reduced recycling of TFRC. Enhanced phosphorylation of M98K-OPTN by TBK1 at S177 plays a crucial role in autophagy-dependent degradation of TFRC leading to cell death.

In normal cells when wild-type OPTN is present, TFRC trafficking and recycling is mediated by OPTN to maintain homeostasis and only a small fraction of TFRC is delivered to autophagosomes where it is degraded. M98K polymorphism alters the function of OPTN in such a way that the recycling of TFRC is reduced and delivery to autophasgosomes is enhanced leading to enhanced degradation of TFRC (48). Thus, OPTN seems to act as a molecular switch to maintain the balance between recycling of TFRC and its delivery to autophagic pathway. M98K mutation alters this balance toward autophagic pathway (Figure 4).

How does M98K mutation alter the properties of OPTN? Compared to wild-type OPTN, M98K-OPTN shows enhanced phosphorylation at S177 in RGC-5 cells. This phosphorylation, which is involved in autophagy, is mediated by TBK1 that is activated by M98K-OPTN (49). Interestingly this activation of TBK1 by M98K-OPTN and enhanced phosphorylation of M98K-OPTN at S177 is seen in RGC-5 cells but not in HeLa or IMR32 cells. Interaction of M98K with TBK1 was not increased but decreased as determined by immunoprecipitation. Mutation of UBD in M98K (M98K-D474N) results in complete loss of activation of TBK1. These observations suggest that interaction of OPTN with TBK1 regulates its activity and functional UBD is required for activation of TBK1 by M98K-OPTN (49). Mutational analysis revealed that phosphorylation of M98K at S177 is required for induction of cell death in RGC-5 as well as 661W cells.

### H486R and Other Mutations

H486R mutation leads to loss of interaction of OPTN with CYLD, which results in impaired NF-κB regulation induced by TNFα, IL-1β, and LPS (60, 61). However, the transgenic mice expressing H486R-OPTN did not show loss of RGCs or any alteration in the retina (77). H486R and some other mutants, H26D, T202R, E322K, did not induce cell death in RGC-5 or 661W cells (48, 86, 92), suggesting, therefore, that these mutants employ indirect mechanisms (e.g., glial cell activation) to contribute to pathogenesis of glaucoma. Since these mutations are rare, an alternate possibility is that these mutants alone may not be able to cause RGC death or glaucoma, and require cooperation from other genetic alterations to cause the disease. Yet, another possibility for the H486R-OPTN is that it may induce glaucoma by causing defects in the immune system. This hypothesis is consistent with the findings that OPTN plays a role in regulating various signaling pathways involved in immune response (7, 64, 87, 88). Since humans differ from mice in their immune system considerably, the H486R may not induce the same alterations in transgenic mice as in humans. Humans have a much longer life span due to which they are expected to encounter more stress than mice in their lifetime, and various types of stress may contribute to age-related glaucoma pathogenesis.

An insertion of 2 base pairs (691_692 ins AG) was reported to be associated with NTG (1). This is perhaps the only mutation of OPTN known so far that is associated with glaucoma as well as ALS (98, 99). This mutation causes a frameshift leading to a premature stop codon that leads to production of a smaller protein of 148 amino acids including 21 aberrant amino acids. This protein localizes in the nucleus and induces apoptosis in retinal cells (99). The mechanism by which this mutant induces apoptosis or glaucoma pathogenesis is not known.

## Autoimmunity and Glaucoma

Glaucoma is a multi-factorial disease and its pathogenesis mechanism is not completely understood. Several mechanisms have been proposed that may contribute to neurodegeneration in glaucoma, such as mitochondrial dysfunction, oxidative stress, secretion of toxic molecules by glial cells, and autoimmunity (7, 64, 100, 101). Alterations in the level of autoreactive antibodies was first reported two decades back, when it was shown that HSP60 antibody level was increased in the serum of glaucoma patients (102). Since then increase or decrease in the level of antibodies against various proteins such as HSP70, HSP27, crytallins, neuron-specific enolase, GST, αFodrin, GFAP, MBP, etc., from sera of NTG as well as POAG patients, has been reported (101). Some of these antibodies induce death of primary RGCs *in vitro*, such as HSP27 and HSP60 antibodies (101, 102). On the other hand, antibodies against some of the proteins, like GFAP, enhance RGC survival and are present at lower level in glaucoma patients (103).

The role of some of the autoreactive antibodies in glaucoma pathogenesis has been explored by immunization of rats with antigens. It was found that rats immunized with HSP27 or HSP60 showed RGC loss and glaucoma-like pathology. In addition, these rats showed complex alterations in autoantibody pattern (104). Immunization with proteins unrelated to glaucoma, such as keratin, did not result in glaucoma or RGC loss, whereas immunization with optic nerve antigens resulted in RGC loss and deposits of IgG in RGC layer (105).

Are altered autoantibody levels causatively associated with glaucoma? The role of autoimmunity in inducing RGC death and glaucoma pathogenesis is still controversial, although several studies indicate its involvement in glaucoma pathogenesis. Autoreactive antibodies are believed to have protective effect on various cells, and are considered as regulatory molecules that are possibly involved in immune regulation and cellular homeostasis (106). These might be involved in attenuating neurodegeneration and, therefore, may contribute to reduction in glaucoma-associated damage. Decrease in cytoprotective autoantibodies and increase in damaging antibodies may contribute to initiation or progression of RGC degeneration associated with glaucoma.

Do mutations in OPTN affect autoimmunity? The H486R mutant is impaired in regulating NF-κB, and NF-κB deregulation is involved in autoimmunity and glaucoma. NF-κB p50-deficient mice show age-related glaucoma phenotype such as RGC loss, excavation of optic nerve head, activation of glial cells, and increase in GFAP level. Interestingly, these mice show autoreactive antibodies against some retinal proteins, indicating involvement of NF-κB mediated autoimmunity in glaucoma pathogenesis (57). Whether H486R mutation in OPTN causes autoimmunity or not, is not known. The E50K-OPTN transgenic mice show GFAP increase and gliosis but presence of autoreactive antibodies has not been tested (78). Several OPTN interacting proteins are involved in immune response and/or NF-κB regulation such as CYLD, A20, UXT, and IK cytokine. The E50K and H486R mutants show reduced interaction with UXT, an NF-κB regulator, and IK cytokine, but the functional significance of these altered interactions is not known (40). The E50K and M98K mutants show altered interaction with TBK1, a kinase involved in immune response (49, 78, 89). The altered interactions of glaucoma-associated OPTN mutants with proteins involved in immune response and/or NF-κB signaling raises the possibility of involvement of autoimmunity in glaucoma pathogenesis by these mutants, which is yet to be explored.

## Potential Therapeutic Directions

TBK1 protein kinase shows enhanced interaction with E50K-OPTN as compared with WT OPTN (78, 89). Therefore, a TBK1 inhibitor amlexanox was tested in transgenic mice as a potential drug. Amlexanox, which is known to inhibit TBK1 and IKKε selectively, is an approved drug for the treatment of bronchial asthma, allergic rhinitis, and conjunctivitis in Japan and elsewhere (7). High dose of this drug in mice resulted in reduction of symptoms of glaucoma in E50K transgenic as well as knock-in mice (7). Inhibition of TBK1 activity by a chemical inhibitor BX795 drastically reduces M98K-induced cell death in RGC-5 as well as 661W cells (49, 86). Surprisingly M98K-induced cell death was inhibited more strongly than E50K-induced cell death. These observations suggest that TBK1 is a potential drug target for M98K-OPTN associated glaucoma also. The GAP protein TBC1D17 is a potential drug target for E50K-OPTN induced glaucoma, because it mediates E50K-OPTN induced death of retinal cells (47). However, this needs to be explored further in a transgenic animal model.

Altered autophagy is involved in mediating retinal cell death induced by E50K and M98K mutants of OPTN. Therefore, manipulation of autophagy is likely to have potential for preventing RGC death associated with glaucoma. Rapamycin, an inducer of autophagy has been shown to inhibit E50K-OPTN induced retinal cell death, whereas chloroquin, an inhibitor of autophagy prevents M98K-OPTN induced retinal cell death.

## Concluding Remarks and Future Directions

The role of OPTN as an adapter protein to mediate various cellular functions such as autophagy, vesicle trafficking, and signaling has been established. However, some questions need to be addressed and the mechanisms are not yet completely understood. Does OPTN mediate autophagy of damaged mitochondria and protein aggregates under physiological conditions? Are some of the functions of OPTN, such as regulation of NF-κB, cell type dependent? Although OPTN is expressed in several tissues, glaucoma-associated mutants do not seem to induce any pathology/disorder in any other cells or tissues in humans except in the retina. This cell type specificity is also seen in cell culture where E50K and M98K mutants induce apoptotic death selectively in RGC-like cells. Similarly, an ALS-associated mutant, E478G-OPTN, induces cell death in a motor neuron-like cell line but not in RGC-like cells. Molecular basis of this cell type specificity of OPTN mutants is a challenging and interesting question, which needs to be addressed. How other mutants of OPTN that do not induce death of retinal cells in culture, contribute to glaucoma pathogenesis, is yet to be investigated. Autophagy appears to play an important role in E50K and M98K induced cell death although the effects of these mutants on autophagy are different. This indicates that OPTN-mediated autophagy is critical for the survival of retinal cells. What is the connection at molecular level between impaired autophagy and cell death by apoptosis? In other words, how impaired autophagy leads to initiator caspase activation? The TBK1 protein kinase has emerged as a potential drug target for E50K-OPTN induced glaucoma. The utility of these TBK1 inhibitors may be explored for M98K-OPTN associated glaucoma also because M98K-OPTN induced retinal cell death is inhibited strongly by a TBK1 inhibitor. Since OPTN has a role in immune signaling pathways, the involvement of OPTN and its mutants in autoimmunity associated with glaucoma needs to be explored.

## Author Contributions

GS and ZS wrote the manuscript and approved the final manuscript.

## Conflict of Interest Statement

The authors declare that the research was conducted in the absence of any commercial or financial relationships that could be construed as a potential conflict of interest.
